# Impact of CLSI Break Point Changes Over the Past Decade on Antimicrobial Susceptibility in Gram-Negative Bacteria

**DOI:** 10.1017/ash.2021.115

**Published:** 2021-07-29

**Authors:** Wesley Johnson, David Burgess, Donna Burgess, Sarah Cotner, Jeremy VanHoose, Justin Clark, Katie Wallace

## Abstract

**Background:** Over the past decade, the CLSI has updated susceptibility break points for several antimicrobial agents. The purpose of this study was to evaluate the impact of these changes against gram-negative bacteria at our academic medical center. **Methods:** In this retrospective, IRB-approved study, we collected consecutive, nonduplicate clinical isolates of *Enterobacter cloacae*, *Escherichia coli*, *Klebsiella aerogenes*, *K. oxytoca*, *K. pneumoniae*, and *Pseudomonas aeruginosa* for the past decade (2010–2019) at our academic medical center and 3 adult ICUs. Susceptibility testing was performed using the BD Phoenix automated system. For these isolates, susceptibilities for 7 β-lactams (aztreonam, ceftriaxone, ceftazidime, cefepime, piperacillin/tazobactam, ertapenem, and meropenem) and 2 fluoroquinolones (levofloxacin, ciprofloxacin) were calculated based upon CLSI break points in 2010 and current CLSI break points in 2020. Any change >5% in susceptibility was deemed significant for this analysis. **Results:** In 17.5% of Enterobacteriales isolates tested, at least 1 antimicrobial demonstrated significant decline. Ertapenem was the most commonly affected antimicrobial (45% of the isolates) followed by ceftriaxone (35%) and cefepime (25%). Susceptibilities of aztreonam, ceftazidime, and meropenem were not affected for any of the Enterobacteriales. The most common organism demonstrating a significant impact on change in susceptibility among the Enterobacteriales was *E. cloacae* (41.7% of the time) followed by *E. aerogenes* (20.8%), *K. oxytoca* (12.5%), *K. pneumoniae* (8.3%) and *E. coli* (4.2%). Most of the impact was observed hospital-wide (33.3%), followed closely by the MICU (28.6%), the NSICU (23.8%) and the CVICU (14.3%). For *P. aeruginosa*, the impact of the antimicrobial break-point changes on susceptibility was more pronounced than the Enterobacteriales. Overall, 93.8% of the time there was a significant decline in antimicrobial susceptibility. Each antimicrobial (ciprofloxacin, levofloxacin, meropenem, and piperacillin/tazobactam) demonstrated a significant decline in susceptibility hospital-wide and in each ICU except for the susceptibility of meropenem in the NSICU. **Conclusions:** Changes in break points had a significant impact on the susceptibility of all antimicrobials for *P. aeruginosa* at our institution, both hospital-wide and in the adult ICUs. Although the impact was less for the Enterobacteriales, ertapenem, ceftriaxone, and cefepime demonstrated significant susceptibility changes, especially with *E. cloacae*. Understanding and evaluating the impact of the break-point changes may lead to changes in empiric therapy in other institutions.

**Funding:** No

**Disclosures:** None

